# Middle ear mucosal regeneration by tissue-engineered cell sheet transplantation

**DOI:** 10.1038/s41536-017-0010-7

**Published:** 2017-03-24

**Authors:** Kazuhisa Yamamoto, Masayuki Yamato, Tsunetaro Morino, Hiroaki Sugiyama, Ryo Takagi, Yuichiro Yaguchi, Teruo Okano, Hiromi Kojima

**Affiliations:** 1 Department of Otorhinolaryngology, Jikei University School of Medicine, 3-25-8 Nishi-shinbashi, Minato-ku, Tokyo Japan; 2 Institute of Advanced Biomedical engineering and Science Tokyo Women’s Medical University, 8-1 Kawada-cho, Shinjuku-ku, Tokyo 162-8666 Japan; 3 Department of Otorhinolaryngology, St. Marianna University School of Medicine, 2-16-1 Sugao, Miyamae-ku, Kawasaki city, Kanagawa Japan

## Abstract

The recurrence of cholesteatoma after surgical treatment often occurs as a result of poor mucosal regeneration in the middle ear cavity and mastoid cavity and changes, such as granulation tissue formation, which impair gas exchange in the middle ear cavity. Conventional tympanoplasty often results in a lack of mucosal regeneration in the resected area of the mastoid cavity. In particular, mucosal regeneration in a poorly pneumatized mastoid cavity is extremely difficult. If the middle ear mucosa can be preserved or rapid postoperative regeneration of mucosa on the exposed bone surface can be achieved after middle ear surgery, the results of surgical treatment for otitis media, including cholesteatoma, can potentially be improved and the physiological function of the middle ear can be recovered. To overcome these limitations, we developed a novel treatment method combining tympanoplasty and autologous nasal mucosal epithelial cell sheet transplantation for postoperative regeneration of the middle ear mucosa. In clinical research, we endoscopically removed an approximately 10 × 10 mm^2^ piece of nasal mucosal tissue. Tissue-engineered autologous nasal mucosal epithelial cell sheets were fabricated by culturing the harvested cells in an aseptic environment in a good manufacturing practice-compliant cell processing facility. The cultivated cell sheets were transplanted, during tympanoplasty, onto the exposed bony surface of the attic of the tympanic and mastoid cavities where the mucosa had been lost. We performed this procedure on four patients with middle ear cholesteatoma and one patient with adhesive otitis media. All patients showed favorable postoperative course with no adverse events or complications and the patients’ hearing ability post-transplantation remained good.

## Nose tissue that helps the ear heal

Transplanting a sheet of cells from the nose to the middle ear could improve postoperative prognosis for middle ear inflammatory conditions. Kazuhisa Yamamoto of Jikei University School of Medicine and colleagues in Japan developed a novel surgical technique to prevent postoperative recurrence of chronic middle ear conditions. In adhesive otitis media, the eardrum gets sucked into and stuck in the middle ear space. Cholesteatoma involves abnormal skin growth in the middle ear. Recurrence often follows surgical treatment for these conditions due to delays in regeneration of the middle ear’s mucosal epithelium. The new technique involves transplanting cultured cells from the lining of the nasal cavity to the middle ear during surgery. Five patients treated this way had favorable postoperative outcomes with no adverse effects or complications and satisfactory improvement in hearing levels.

## Introduction

In 2004, The WHO listed acquired hearing loss as a leading disease burden in persons aged ≥ 15 years.^1^ Hearing loss is a major factor that can reduce patients’ quality of life. Adhesive otitis media and cholesteatoma are typical middle ear diseases that lead to hearing loss. In adhesive otitis media, the tympanic membrane cannot maintain its normal position and becomes adhered to the bone wall of the middle ear cavity. As a result, aeration of the middle ear cavity is lost and vibration of the adhered tympanic membrane is hampered, resulting in hearing loss. Progression of this condition can result in cholesteatoma.

In cholesteatoma, keratinized stratified squamous epithelium that has invaded the middle ear destroys the surrounding bone tissue. The destruction of the ossicles can lead to hearing loss. Further invasion of the tissue into the inner ear and associated spread of inflammation can result in irreversible sensorineural hearing loss. Moreover, destruction of the surrounding bone tissue can lead to a variety of additional serious complications, including facial nerve paralysis, dizziness due to destruction of the semicircular canals, and intracranial complications, such as meningitis, brain abscess, etc.

Repeated occurrence of otitis media during childhood can inhibit the development of the mucous membrane of the mastoid and inhibit pneumatization due to reduced gas ventilation capacity. This pathology is said to be one cause of adhesive otitis media and/or cholesteatoma.^2–7^ The normal tympanic cavity and lumen of the mastoid are covered with middle ear mucosa that also functions as a periosteum and has the capacity for gas ventilation. Maintaining middle ear pressure is important for maintaining the mobility of the tympanic membrane necessary for efficient sound conduction and the gas ventilation capacity of the middle ear mucosa is essential for regulation of this middle ear pressure.

There is no curative therapy for adhesive otitis media and cholesteatoma other than tympanoplasty. One of the additional objectives of tympanoplasty is to improve hearing. However, to accomplish this, the middle ear cavity must be aerated so that the tympanic membrane vibrates efficiently and sound can be transmitted to the cochlear via the ossicles.^8^ In order for a normal middle ear cavity to form after surgery, regeneration of the middle ear mucosa, recovery of the physiological gas ventilation capacity, and prevention of tympanic membrane adhesion are essential. However, in the presence of otitis media, middle ear mucosal function is inherently damaged and complicates the creation of an effectively pneumatized cavity, because postoperative regeneration of the middle ear mucosal epithelium is delayed. In particular, in adhesive otitis media patients whose tympanic membrane is retracted and adhered to the inner wall of the middle ear cavity, the epithelium is peeled off during surgery, exposing the bone surface of the middle ear and making it difficult to preserve the middle ear mucosa. For this reason, patients with adhesive otitis media have poor hearing improvement after surgery when compared with patients recovering from other middle ear diseases.

The current operative procedures for cholesteatoma surgery include “canal wall up” tympanoplasty, which preserves the posterior wall of the external ear canal, and “canal wall down” tympanoplasty, which involves removal of the posterior wall. While “canal wall up” tympanoplasty is excellent for maintaining the physiological form of the external ear canal, postoperative recurrence is quite common. Residual cholesteatoma can be prevented by use of an endoscope, but it is difficult to suppress relapse completely. Although “canal wall down” tympanoplasty can prevent recurrence of cholesteatoma, this surgery can compromise the physiology of the external ear canal, which may cause postoperative cavity problems, etc. The ideal operative procedure would preserve the posterior wall of the external ear canal and also form a mastoid cavity with good postoperative pneumatization. However, no such definitive surgical technique exists today and improvement of ordinary tympanoplasty treatment and resulting therapeutic effect is limited.

Considering this context, if regeneration of the damaged middle ear mucosa were possible in the early postoperative period, it would be possible to prevent re-adhesion of the tympanic membrane and recurrence of adhesive otitis media. Additionally, regeneration of middle ear mucosa would prevent recurrence of cholesteatoma on the preserved the posterior wall of the external ear canal. However, achieving early regeneration of the middle ear mucosa has been a major challenge. Currently, a method was reported for where nasal mucosa and oral cavity mucosa are directly transplanted to replace the middle ear mucosa,^9^ presenting a potential effective treatment for preventing postoperative retraction and re-adhesion of the tympanic membrane as well as promoting regeneration of the middle ear mucosa. Transplantion of materials, such as collagen sponges, silicon plates, etc., have also been attempted to protect and encourage the regeneration of the middle mucosa.^10, 11^ The efficacy of those methods and robustness of these techniques, however, have not yet been firmly established.

**Fig. 1:**
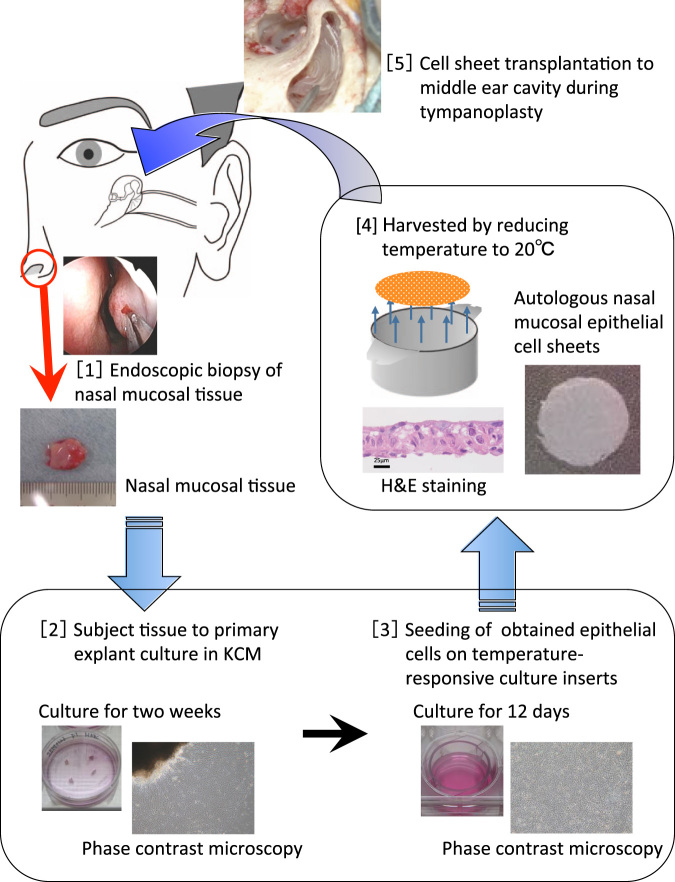
Transplantation of autologous tissue-engineered epithelial cell sheets fabricated from nasal mucosa. [1] Infiltration anesthesia of the inferior nasal turbinate mucosa was performed via nasal endoscopy by application of xylocaine. A *ca*. 10 × 10 mm section was harvested from the inferior nasal turbinate mucosa. [2] The harvested epithelia were minced as finely as possible, placed in a culture dish coated with type I collagen and subjected to primary explant culture in KCM for 2 weeks. Cells started to grow and move outside from the explant of nasal mucosa after 3 days of culture. [3] The cultured cells were trypsinized and then seeded on a temperature-responsive cell culture insert and cultured for 12 days in KCM. Epithelial cells had a polygonal cobblestone shape after just 2 passages. [4] Cultured epithelial cells on the temperature-responsive culture insert were easily harvested as an intact contiguous sheet by simply reducing the temperature of the culture dish to 20 °C for 30 min, without requiring any enzymatic treatment. [5] After tympanoplasty was performed, the cell sheets were transplanted onto the middle ear cavity and mastoid cavity sites with loss of mucosa using a microscope

To date, we have demonstrated the ability of transplanted cultured nasal mucosal epithelial cell sheets to promote regeneration of the middle ear mucosa in rabbits^12^ and we have succeeded in producing nasal mucosal epithelial cell sheets in humans.^13^ Based on these results, we have developed a new surgical technique that combines autologous transplantation of cultured epithelial cell sheets with tympanoplasty to promote postoperative regeneration of the middle ear mucosa (Fig. 1). Here, we present the clinical findings of our objective evaluation of the safety and clinical efficacy of this new treatment method.

The primary endpoint was evaluation of the safety of this new therapeutic technique (i.e., manifestation of adverse reactions, the presence/absence of abnormal changes in hematology test values at 1 month post-transplantation, and the presence/absence bleeding from the nasal cavity after tissue harvest). In addition, the therapeutic efficacy was established on the basis of postoperative tympanic membrane findings, computed tomography (CT) findings, and pure-tone audiometry taken after more than 6 months had passed since transplantation.

## Results

### Subjects

We obtained formal approval to initiate this clinical study from the ethics committee of our institution and conducted the study according to the Guidelines on clinical research using human stem cells outlined by the Ministry of Health, Labour, and Welfare. This trial is registered with the JMA Clinical Trials Registry as No. JMA-IIA00193. This is the first clinical study of its type in the world.

The clinical subjects were five patients (two males, three females) who were diagnosed with adhesive otitis media or cholesteatoma between January 2014 and May 2015. The diagnoses were pars flaccida cholesteatoma in two patients, pars tensa cholesteatoma in two patients, and adhesive otitis media in one patient. The selection criteria were for patients (1) diagnosed with cholesteatoma otitis media or adhesive otitis media requiring tympanoplasty, (2) of at least 20 years of age, and (3) granting oral and written informed consent. Patients with a serious underlying disease (immunodeficiency, heart disease, kidney disease, liver disease, diabetes, asthma, etc.), an infectious disease, a malignant tumor or nasal sinus disease were excluded. Table 1 presents a breakdown of the cases. Autologous transplantation of nasal mucosal epithelial cell sheets was performed at the time of tympanoplasty. At the time of tympanoplasty, the age range of the patients was 28–61 years (mean: 46.6 years) and the post-transplantation observation period ranged from 12–27 months (mean: 18 months).

**Table 1:** Preoperative characteristics of patients with cell sheet transplantation

Patient no.	Age/gender	Diagnosis	Extent of cholesteatoma invasion	Hearing level (dB)
1	28/Female	Pars flaccida cholesteatoma	Reaching the mastoid	23.3
2	61/Female	Pars tensa cholesteatoma	Reaching the attic	56.7
3	38/Male	Pars tensa cholesteatoma	Reaching the attic	26.7
4	54/Male	Pars flaccida cholesteatoma	Reaching the mastoid	53.3
5	52/Female	Adhesive otitis media		61.7

### Characterization of tissue-engineered nasal mucosal epithelial cell sheets

**Fig. 2:**
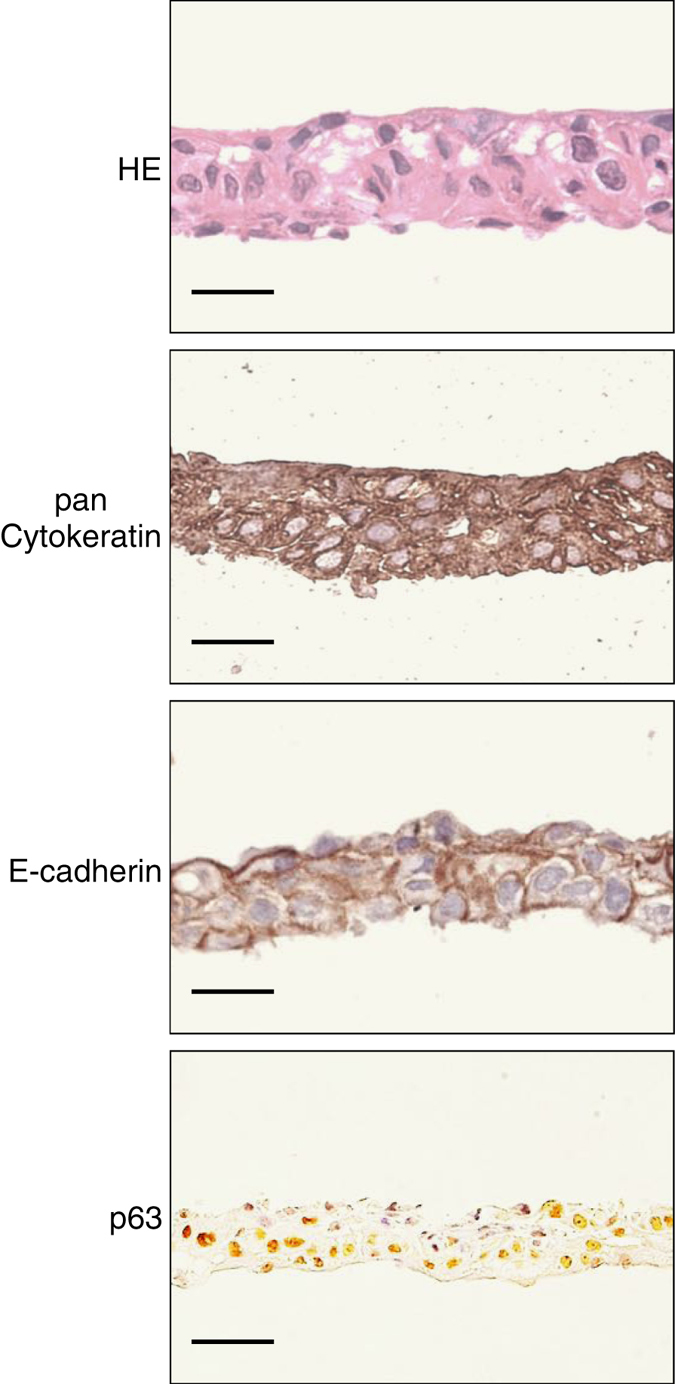
Histological and immunohistochemical analyses of nasal mucosal epithelial cell sheets. Nasal mucosal epithelial sheets were subjected to paraffin-embedded sectioning and stained with HE (first row), anti-pan cytokeratin (second row), anti-E-cadherin (third row) and anti-p63 (fourth row). The HE-stained specimens showed that fabricated cell sheets consisted multi-layered mucosal epithelial cells. All epithelial cell layers of the fabricated cell sheets expressed pan-cytokeratin. E-cadherin was confirmed to be expressed between the epithelial cells in the cell sheets. The epithelial cells in the cell sheets also expressed p63. Bars indicate 25 µm

The prepared nasal mucosal epithelial cell sheets had a structure consisting of several epithelial cell layers, all of which were pan-cytokeratin positive. Keratinocytes are known to express E-cadherin, which are cell–cell adhesion proteins found in adherens junctions. E-cadherin expression was also detected in the nasal mucosal epithelial cell sheets. Expression of p63, a marker of epithelial stem cells, was also seen in the cell sheets (Fig. 2).

The quality tests were performed using the culture supernatants of the cell sheets prior to transplantation, and all fabricated cell sheets satisfied the prescribed standard. In addition, the prepared cell sheets showed an average total cell count of 3.8 ± 2.2 × 10^5^ cells/sheet, survival rate of 88.5 ± 0.04%, and pan-CK-positive cell rate of 90.8 ± 0.06%. The cell sheets produced for all of the patients met the preset standard values (Table 2).

**Table 2:** Results of the quality control tests of cell sheets one day before transplantation

	Patient number				
	1	2	3	4	5
Cell number (cells/sheet)	3.1 × 10^5^	1.7 × 10^5^	6.3 × 10^5^	6.7 × 10^5^	1.2 × 10^5^
Cell survival rate (average %)	82.7%	85.0%	90.7%	91.7%	92.3%
PCK-positive cells (%)	85.9%	80.5%	96.9%	96.6%	94.2%
Mycoplasma pneumoniae	Negative	Negative	Negative	Negative	Negative
Endotoxin (EU/mL)	0.14	<0.008	0.98	2.84	3.97
Bacteria and fungi	Negative	Negative	Negative	Negative	Negative
Virus (HBV, HCV, HIV-1, HTLV-1)	Negative	Negative	Negative	Negative	Negative

### Adverse events (AEs) and complications

None of the patients showed abnormal changes in any of the blood test (complete blood count and blood chemistry) parameters after the transplantation and there were also no AEs or complications. Additionally, none of the patients experienced renewed bleeding from the nasal cavity mucosa donor site following hemostasis and the donor sites showed good mucosal epithelization.

### Clinical results of transplantation of the cell sheets

**Fig. 3:**
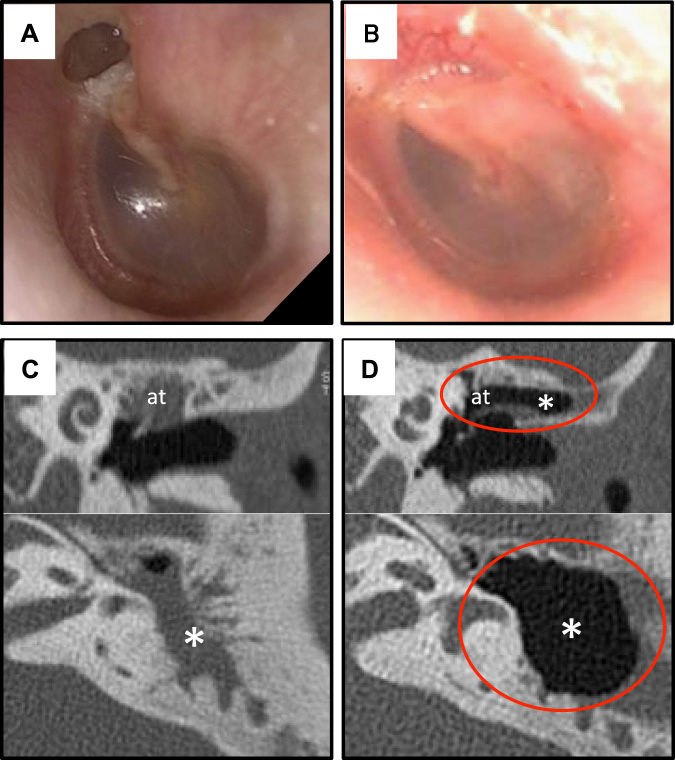
Photographs and CT images of the tympanic membrane and temporal bone before and after cell sheet transplantation (case 1). **a** Image of the tympanic membrane before surgery. There is retraction in the attic of the tympanic membrane. The image shows pars flaccida cholesteatoma. **b** An image of the tympanic membrane 12 months after surgery shows a favorable surgical outcome, without recurrence. **c** Preoperative CT images of the tympanic cavity and mastoid cavity: the upper image is the coronal view and the lower image is the axial view. Pneumatization is inhibited. There is no air (*black space*) visible in the attic (at) of the tympanic cavity or mastoid cavity (*). **d** CT images 12 months after surgery: the upper image is the coronal view and the lower image is the axial view. There is a well-pneumatized space in the region between the attic of the tympanic cavity and the mastoid cavity (indicated by *red* circles). CT: computed tomography

The tympanic membrane findings at 6 months after transplantation did not show any retraction of the attic or recurrence in patients with pars flaccida cholesteatoma. The patients with pars tensa cholesteatoma showed no retraction of the pars tensa, no adhesion, and no recurrence. The cases of adhesive otitis media showed no evidence of re-adhesion of the tympanic membrane. All of the patients showed good tympanic membrane morphology after the transplantation. None of the cholesteatoma patients showed residual cholesteatoma. The CT findings at one year post-transplantation showed pneumatization of the mastoid cavity in 3 of 4 cholesteatoma patients who had undergone mastoidectomy and canal wall up tympanoplasty. The CT at 3 months after transplantation revealed pneumatization at the cell sheet transplantation site from the attic and the CT at 6 months showed the mastoid cavity to be completely pneumatized (Fig. 3).

Ossiculoplasty was performed in all of the patients and consisted of reconstruction of the columella on the stapes in four patients and reconstruction of the columella on the foot plate in 1 patient. The mean hearing level of the patients was 55.4 dB, preoperatively and 33.3 dB, postoperatively. The postoperative air–bone gap was within 10 dB for 1 ear, 11~20 dB for three ears, and greater than 31 dB for one ear (Table 3).

**Table 3:** Results of the clinical study in five patients who received transplants of tissue-engineered autologous nasal mucosal epithelial cell sheets

Patient number	Surgical method	Type of ossiculoplasty	Re-retraction or re-adhesion of tympanic membrane	Postoperative aeration		Recurrence of cholesteatoma	Postoperative hearing level (dB)	Postoperative air-bone gap (dB)	Complications	Months of follow-up
				attic	mastoid					
1	Canal wall up tympanoplasty	Columella on the stapes	None	+	+	None	18.3	10.0	None	27
2	Canal wall up tympanoplasty	Columella on the stapes	None	+	+	None	35.0	20.0	None	20
3	Canal wall up tympanoplasty	Columella on the stapes	None	+	+	None	25.0	15.0	None	16
4	Canal wall up tympanoplasty	Columella on the stapes	None	+	−	None	31.7	15.1	None	14
5	Tympanoplasty without mastoidectomy	Columella on the footplate	None			None	56.7	38.7	None	12

## Discussion

To date, the canal wall up tympanoplasty has been necessary following mastoidectomy for cholesteatoma and has generally been performed using an ordinary surgical drill. However, since bone is drilled into the lesion, including normal middle ear mucosa, at the time of removal of cholesteatoma in this procedure, in most cases, it is difficult to preserve normal mucosa. Following conventional tympanoplasty, mucosa is often not regenerated in the mastoid cavity, which leads to increased granulation throughout the cavity, including on the exposed bone surface. The main cause of recurrence of cholesteatoma is this inhibition of mucosa regeneration in the middle ear and mastoid cavity, resulting in damage to the gas-exchange function in the middle ear cavity due to increased granulation In particular, without early regeneration of postoperative mucosa in the attic, recurrence of cholesteatoma is triggered. Thus, to prevent recurrence of cholesteatoma, early postoperative regeneration of mucosa, especially in the attic, is essential. However, technically, drilling the bone of the attic is unavoidable when removing cholesteatoma, making preservation of the attic mucosa impossible in most cases.

Regenerative medicine by the cell sheet technique using temperature-responsive culture dishes^14, 15^ has already shown clinical success in treatment of limbal stem cell deficiency^16^ and artificial esophageal ulcerations after endoscopic mucosal resection.^17^ We confirmed the effectiveness of transplantation of cultured autologous nasal mucosal epithelial cell sheets in an animal model of middle ear mucosal damage.^12^ Rabbit nasal mucosal epithelial cell sheets were fabricated on a temperature-responsive culture dish, and transplanted into the damaged middle ear of rabbit, which was surgically created. The healing of middle ears was evaluated by histology and X-ray computed tomography after transplantation. Functional evaluation was performed by measuring the maximum middle ear total pressure reflecting a trans-mucosal gas exchange function. Two control groups were used: the normal control group and the mucosa-eliminated control group. Transplantation of cell sheets suppressed the bone hyperplasia and the narrowing of pneumatic space in the middle ear cavity compared with the mucosa-eliminated control group. The mucosal gas exchange function was also better in the cell sheet-transplanted group. Nasal mucosal epithelial cell sheet was confirmed to be useful as an effective graft material after middle ear surgery. Moreover, we showed that it was possible to prepare human autologous nasal mucosal epithelial cell sheets.^13^ These successful applications of this method led us to initiate the present study.

We previously prepared middle ear mucosal epithelial cell sheets in an experimental animal model and investigated the ability of those sheets to promote mucosal regeneration when they were transplanted into the middle ear.^18–20^ However, when considering clinical application of this method, various logistic and ethical issues had to be overcome. Middle ear mucosal epithelial cells are not as easy to culture as other epithelial cells and it is difficult to harvest a sufficient amount of normal middle ear mucosa needed to prepare middle ear mucosal epithelial cell sheets in cell culture.^13^ Additionally, it would be difficult to perform this resection as an outpatient procedure, requiring an additional separate surgery to harvest the cells needed for creating the cell sheets, thus increasing the surgical burden on the patient. Further, harvesting tissue from the patient’s healthy ear poses the risk of harming that ear’s hearing ability. It is not rare for adhesive otitis media and/or middle ear cholesteatoma to be bilateral,^21, 22^ which would limit applicability of this therapy in patients, arguably with the most need, as they would not have healthy middle ear mucosa available for harvest. These difficulties complicated the clinical application of our cell sheet transplantation technique.

In order to overcome these problems, we focused on use of the nasal mucosa as a cell source for preparing the cell sheets. The nasal cavity is anatomically continuous with the middle ear cavity through the eustachian tube, and embryologically, the nasal mucosa, like the middle ear mucosa, belongs to the respiratory epithelium.^23^ Moreover, we confirmed that the middle ear mucosa and nasal mucosa are also very similar histologically.^13^

In considering clinical application in humans, the nasal mucosa, unlike the middle ear mucosa, is safe and easy to harvest in an outpatient procedure. It is also possible to collect a sufficient amount of tissue for culture. We chose the inferior turbinate mucosa that we can harvest most safely and easily with good cell proliferation ability. There is no bleeding from the nasal cavity after tissue harvest and there are no other AEs from this procedure. Further, the mucosal defect at the nasal cavity donor site shows good epithelialization and no problems are generated at the donor site. The otolaryngologist who performs cell sheet transplantation can also harvest the nasal mucosa used as the cell source. Additionally, the culture of the nasal mucosa epithelial cells is relatively easy, making this cell source ideal for cell sheet preparation. Since the cell sheets can be made without the use of feeder cells, only autologous cells are used, and the patient does not have the risk of developing an immune rejection reaction. Thus, implementing nasal mucosa overcomes many of the limitations that existed in use of middle ear mucosa for cell sheet construction.

Besides bleeding from the nasal cavity after tissue harvest, one of the probable AEs is that the cell sheet doesnot survive and transplanted cells cause necrosis after transplantation. In such case, the granulation tissue might be developing in the mastoid cavity. As a result, the post-transplant situation is considered to become same to the conventional postoperative situation. In addition, since the potential cell source is the differentiated nasal mucosal epithelial cell, it is difficult to assume that the fabricated cell sheet has tumorigenicity.

It was reported that harvested autologous nasal mucosal tissue can be directly transplanted immediately following collection, such as into the middle ear cavity.^9^ However, since the subepithelial layer of the nasal mucosa is a glandular tissue, there is a possibility that after transplantation, secretions from the glands could be retained in the middle ear and cause otitis media with effusion. In addition, since nasal mucosal tissue has a very thick subepithelial layer of tissue,^13^ efficient transplantation into the narrow middle ear cavity would be an extremely difficult procedure. Additionally, the bone surface at the transplantation site has inherently poor blood circulation and is inadequate as a scaffold for tissue engraftment. Thus, sustained engraftment of the nasal mucosal tissue is difficult to accomplish in the direct transplantation of the harvested mucosal tissue.

The cultured nasal mucosal epithelial cell sheet is composed of epithelial cells without subepithelial or glandular tissue,^13^ meaning that there is no potential to cause otitis media with effusion. The cultured nasal mucosal epithelial cell sheet is also similar to the native middle ear mucosa in width, making it suitable for easy transplantation into the middle ear cavity. Since the cell sheet retains cell–cell adhesion and deposited extracellular matrix it can be expected to exhibit good engraftment to the tissue at the graft site.

The nasal mucosa cell sheet is formed from epithelial cells and, like the inherent physiological middle ear mucosa, it is very thin and not elastic. As a result, it could be somewhat difficult to insert the cell sheet into narrow sites, such as the attic, and ensure that it attaches with suitable tension. However, with the device that we used in this study, the surgeon is able to easily, reliably, and smoothly deliver a cell sheet into the attic, overcoming the poor manipulability of the cell sheet and the narrow anatomical features of the middle ear cavity (device and methods of transplantation are mentioned later in the section of the materials and methods).

The overall recurrence rate following canal wall up tympanoplasty, the conventional method for treating cholesteatoma while preserving the posterior wall of the external ear canal, has been reported to be 15–61% (refs 24–29). In our present clinical study, recurrence was not seen in any of the four cholesteatoma patients at least twelve months follow up receiving our autologous cell sheet transplantation treatment. The rate of pneumatization following mastoidectomy is typically reported to only be *ca*. 40% (refs 30, 31), but we found pneumatization extending up to the mastoid cavity in 3 of 4 (75%) of our patients. These results indicate that the grafting of autologous cell sheets to the bone surfaces exposed after surgery led to early regeneration of the mucosa, which in turn, brought about recovery of middle ear physiological function and was effective in preventing recurrence of tympanic membrane adhesion and cholesteatoma.

In this study, the percentage of cytokeratin-positive cells was quantitatively analyzed by flow cytometry and the results showed that the purity of epithelial cells in the cultured cell sheets was higher than 80%.We considered the epithelial cells were main population of cell sheets because the all layers of fabricated cell sheets expressed pan-cytokeratin in this study. Although it is necessary to investigate whether the transplanted cells remain in future, in previous animal study, we could observe the cells with epithelioid layer structure at the cell sheet transplanted site at 8 weeks after transplantation, and we concluded these cells were the transplanted cells. Although the rate of existence of stem/progenitor cells was unknown, in this clinical study, the transplanted cells within the sheets also have a potential to survive in the host middle ear cavity and become a part of the functioning tissue.

On the other hand, it is known that the cell sheets provide a paracrine function in the other cell sheet therapies.^32–34^ Although it is currently unclear whether the source of the therapeutic cytokines is the transplanted cells or native middle ear mucosal cells, there is also a possibility that this nasal mucosal epithelial cell sheet amplified subsequent paracrine effects to recover/regenerate the damaged residual middle ear mucosa.

Based on these considerations, we suggested that establishment of early communication between the cell sheets and host middle ear cavity boost postoperative middle ear mucosal regeneration.

It remains to be determined whether the primary benefits of cell-sheet transplantation are a consequence of (1) an effect of functioning tissue by contributing directly to middle ear mucosal regeneration, (2) paracrine effects emanating from the cell sheet, or (3) a combination of these effects.

These are future research issues, however, the results showed that the cell sheet have a potential to enhance postoperative regeneration of middle ear mucosa and improve therapeutic outcomes, compared with conventional tympanoplasty.

From the viewpoint of postoperative hearing results, this study shows that a novel method combining canal wall up tympanoplasty and cell sheet transplantation works as well as, or better than, conventional tymapnoplasty.^35, 36^ Accordingly, not only the results of recurrence prevention of cholesteatoma but also the postoperative hearing results were good. Moreover, the cell sheet transplantation was not seen to lead to any AEs or complications, thus confirming the safety of this grafting technique.

Currently the follow-up period is still short and the number of patients was small. Long-term follow-up and experience with a large series of patients are needed to assess further the benefits and risks of this method, which offers the potential to treat many patients with middle ear disease.

Since there is currently no method for reliably achieving postoperative mucosa regeneration, the efficacy shown by our present method of autologous cell sheet transplantation is truly groundbreaking. We surmise that these promising clinical results where obtained due to the ability of the autologous nasal mucosa epithelial cell sheets to engraft to the exposed sites where they were transplanted, allowing them to become a part of the functioning tissue and amplify subsequent paracrine effects in promoting postoperative mucosal regeneration.

## Conclusion

To our knowledge, this clinical research is the world’s first-in-human study to transplant cultured cells into the human ear. This study represents a great step forward in the development of a new surgical approach for treating adhesive otitis media and cholesteatoma. This treatment simultaneously preserves the external ear canal morphology, as in standard canal wall up tympanoplasty, and incorporates autologous cell sheet transplantation, which enables prevention of recurrence of cholesteatoma, tympanic membrane adhesion, and tympanic membrane retraction.

The incorporation of cell sheet transplantation into the conventional operative procedure has the potential to bring about significant improvement in the prognosis for patients undergoing middle ear surgery. To date, surgery for middle ear cholesteatoma patients has a high risk of recurrence, necessitating further surgical treatment. However, through this single-surgery cell sheet transplantation technique, postoperative regeneration of the middle ear cavity mucosa and pneumatization could be ensured and recurrence of the previous pathology could be significantly reduced, limiting the need for further surgery. This treatment method reduces the overall invasiveness of the surgical treatments required and improves patient prognosis in terms of recurrence and postoperative hearing ability. We anticipate that the novel technique described here will become a new treatment for prevention of recurrence of adhesive otitis media and middle ear cholesteatoma, providing relief for long-suffering patients.

## Materials and methods

### Preparation of culture medium

Autologous keratinocyte culture medium (KCM) was prepared for each patient, consisting of a basal mixture of three parts Dulbecco’s modified Eagle’s medium (DMEM; Sigma) and one part nutrient mixture Ham’s F-12 medium (Sigma), supplemented with 5% autologous human serum, hydrocortisone (0.5 µg/mL), insulin (5.0 µg/mL), transferrin (10 µg/mL), triiodothyronine (6.5 ng/mL), epidermal growth factor (0.5 ng/mL), cholera toxin (1 nM), penicillin G sodium (100 U/mL), and streptomycin sulfate (100 mg/mL). Before preparation of patients’ serum for (KCM), each patient’s blood was screened to ensure that he/she had no infections.

### Preparation of nasal mucosal epithelial cell sheets

Inferior nasal turbinate mucosa was harvested by nasal endoscopy as an outpatient procedure. Infiltration anesthesia was performed by application of xylocaine (AstraZeneca, Osaka, Japan) to the inferior nasal turbinate, and a *ca*. 10 × 10 mm section was harvested from the inferior nasal turbinate mucosa (Fig. 1[1]). Hemostasis was performed at the turbinate mucosa donor site. The harvested nasal mucosa was placed in DMEM containing penicillin G sodium (100 U/mL) and streptomycin sulfate (100 mg/mL) and immediately transported to a good manufacturing practice-compliant cell processing facility (CPF).

All procedures were carried out under aseptic conditions at the CPF. Each nasal biopsy specimen was sterilized once with povidone-iodine and three times with DMEM containing penicillin G sodium (100 U/mL) and streptomycin sulfate (100 mg/mL). Each sterilized nasal biopsy specimen was then divided into quarters and treated with 1000 U/mL of dispase (Godo Shusei, Tokyo, Japan) for 2 h at 37 °C. The epithelia were separated from the underlying substantia propria, minced as finely as possible, placed in a culture dish coated with type I collagen (BD BioCoat, Franklin Lakes, NJ, USA), and subjected to primary explant culture in KCM (Fig. 1[2]). After 2 weeks, the cultured cells were trypsinized and seeded on a temperature-responsive cell culture insert (CellSeed, Tokyo, Japan) at a density of 5 × 10^4^ cells/cm^2^ (Fig. 1[3]). After 12-day cultivation in KCM, the cultured cells were harvested from the insert by reducing the temperature from 37 °C to 20 °C for 30 min (Fig. 1[4]).

Prior to the scheduled day of transplantation, the prepared autologous cell sheets were subjected to various quality tests. Using the culture supernatants, sterility tests, mycoplasma tests, virus tests (HBV, HCV, HIV, HTLV), syphilis tests, and endotoxin tests were performed. Additional cell sheets were cultured for each patient so that the cell count, viability, and the cell purity (confirmed by measuring the pan-CK-positive cell rate by flow cytometry (MACSQuant^®^ Analyzer, Miltenyi Biotec, Germany) could also be evaluated the day before the scheduled transplantation. Cell sheets for each patient were determined to meet the threshold values for each of these quality tests.

Based on criteria of previous clinical studies,^16, 17^ the prescribed criteria were the cell count > 1.0 × 10^5^, viability (the survival rate) > 70%, the cell purity > 70%, endotoxin < 5.0 EU/mL, sterile, endotoxin < 0.5 EU/mL, and absence of mycoplasma, virus, and syphilis.

### Immunohistochemistry

For cross-sectional analysis, the nasal mucosal epithelial cell sheets were fixed with 10% neutral buffered formalin and routinely processed into 3 µm-thick paraffin-embedded sections. Hematoxylin and eosin (HE) staining was performed by conventional methods. For immunohistochemistry, de-paraffinized sections were washed with phosphate-buffered saline and digested with protease K (DakoCytomation, Glostrup, Denmark). Sections were then treated with each of the following antibodies according to the manufacturers’ suggested protocols: mouse monoclonal anti-pancytokeratin (1:20 dilution, AE1/AE3, Abcam, Cambridge, UK), mouse monoclonal anti-E-cadherin (1:100 dilution, NCH-38, DakoCytomation) and anti-p63 (1:100 dilution, V9, DakoCytomation).

### Tympanoplasty

For each of the patients, tympanoplasty was performed via a postauricular approach under a microscope (OPMI^®^ Vario/S88, ZEISS, Germany). Mastoidectomy was performed for all patients with cholesteatoma and the cholesteatoma was removed by canal wall up tympanoplasty. An endoscope (HOPKINS^®^Telescope, KARL STORZ, Germany) was used to confirm that there was no residual cholesteatoma post-surgery. For the adhesive otitis media cases, the tympanic membrane adhered to the tympanic cavity was peeled off and the adhered pathological tympanic membrane was removed. For ossicular reconstruction, columella on the stapes were performed in patients whose stapes superstructure remained, while columella on the foot plate was performed in patients whose stapes superstructure had been lost.

### Transplantation of cell sheets to the middle ear

In all patients, the transplantation of the prepared autologous cell sheets to the middle ear cavity and mastoid cavity was performed under a microscope (Fig. 1[5]).

**Fig. 4:**
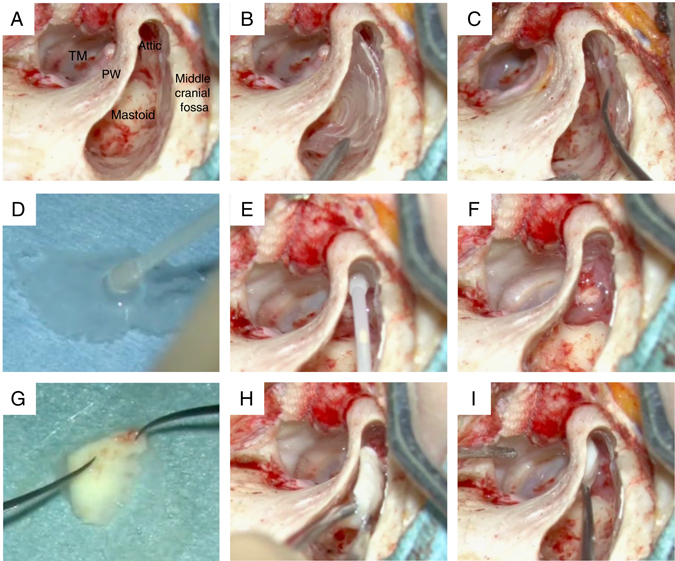
Outline of the various procedures for cell sheet transplantation into the mastoid cavity and attic under a microscope following canal wall up tympanoplasty. **a** Micrograph of the mastoid cavity and attic after canal wall up tympanoplasty in cholesteatoma patients who had undergone mastoidectomy. **b** The cell sheet was delivered into the mastoid cavity on a supportive silicon plate and the surface containing the cell sheet was pressed onto the exposed bone surface in the middle cranial fossa. **c** The silicone plate was removed and the cell sheet was spread onto the exposed bone surface in the middle cranial fossa. **d** Using a device equipped with a 1-mL syringe probe, the central portion of a cell sheet was aspirated, and the sheet was held with the tip of the device. **e** The cell sheet was fully aspirated into the syringe probe tip, the device holding the sheet was placed just outside of and pointed toward the narrow attic, and the cell sheet was extruded in manner resembling an inflated parachute. **f** The extruded cell sheet was spread out as fully as possible to cover the attic bone surface, completing the cell sheet transplantation. **g** A cell sheet was placed on a piece of auricular cartilage harvested for use in scutumplasty. The cell sheet is placed on the surface that becomes the mastoid cavity side of the auricular cartilage. **h** The cartilage combined with the cell sheet was placed into the attic. **i** The cartilage combined with the cell sheet was carefully pressed to the scutum. To prevent recurrence of cholesteatoma, scutumplasty was performed transplanting a hybrid implant composed of a cell sheet covering a piece of auricular cartilage. TM: tympanic membrane, PW: posterior canal wall

For the cholesteatoma patients in whom mastoidectomy and canal wall up tympanoplasty were performed, the cell sheets were transplanted onto the bone surface exposed due to the loss of middle ear mucosa in the mastoid cavity and the attic. In order to graft the cell sheet to the mastoid cavity bone surface, the cell sheet was placed on a silicon plate for support and then attached to the bone surface. Finally, the silicon plate was removed from the cell sheet, leaving the cell sheet alone on the bone surface (Fig. 4a, b, c). Transplantation of a cell sheet to the narrow attic was performed using a device equipped with a 1-mL syringe probe (20 G, length 38 mm, tip diameter 2.0 mm). The recovered cell sheet was aspirated into this device (Fig. 4d) and then extruded onto the grafting site. The cell sheet was then spread out as fully as possible like a parachute (Fig. 4e, f).

**Fig. 5:**
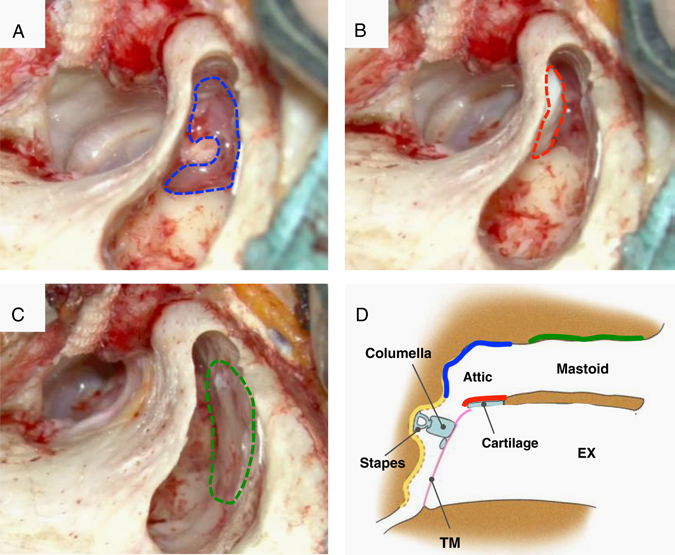
The procedure for cell sheet transplantation following canal wall up tympanoplasty in a patient with left pars flaccida cholesteatoma. **a** A cell sheet that was transplanted into the narrow attic. (The transplanted cell sheet is outlined with *blue dashes*.). **b** Scutumplasty was carried out together with cell sheet transplantation (hybrid scutumplasty). A cell sheet that was transplanted onto the mastoid cavity surface on a piece of cartilage used in the scutumplasty. (The transplanted cell sheet is outlined in *red dashes*.). **c** A cell sheet transplanted onto the exposed bone surface of the middle cranial fossa. (The transplanted cell sheet is outlined in *green dashes*.). **d** Diagram of the coronal cross-section. The cell sheet transplanted to the attic is depicted in *blue*, the hybrid cell sheet-cartilage implant transplanted to the mastoid surface is depicted in *red*, and the cell sheet transplanted to the bone surface of the middle cranial fossa is depicted in *green*. TM: tympanic membrane, EX: external ear canal

For the patients with pars flaccida cholesteatoma, the objective was to prevent recurrence of the cholesteatoma. Hybrid scutumplasty was performed by placing the cell sheet on the surface of the cartilage on the mastoid cavity side (Fig. 4g, h, i). Figure 5 shows the procedure for the cell sheet transplantation performed following canal wall up tympanoplasty in the patient with pars flaccida cholesteatoma.

**Fig. 6:**
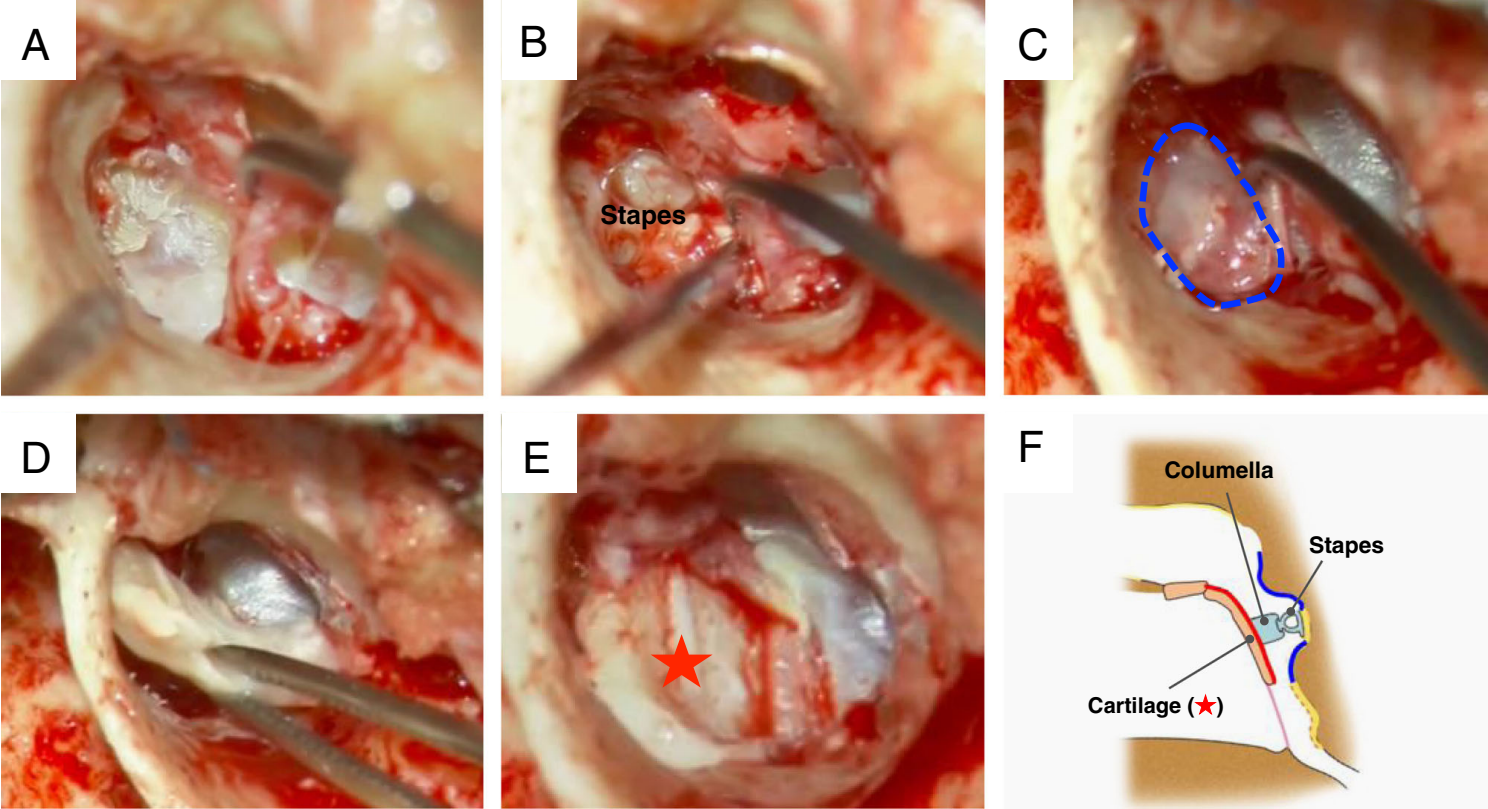
Cell sheet transplantation for the patients with pars tensa cholesteatoma and adhesive otitis media. **a** Epithelium adhered around the stapes. **b** The adhered lesion is completely stripped away and removed. **c** A cells sheet is transplanted around the stapes using the earlier-described device. **d** A hybrid graft material composed of cartilage and a cell sheet is placed onto the transplant site. Tympanoplasty is performed so that the cell sheet faces the tympanic cavity side. **e** The cell sheet is situated on the back surface of the cartilage, as indicated by the *red star*. **f** Diagram of the coronal cross-section. The cell sheet around the stapes is depicted in *blue*, while the cell sheet on the rear surface of the reconstructed tympanic membrane is in *red*. With the objective of preventing re-adhesion of the tympanic membrane, cell sheet transplantation was performed around the stapes and on the rear surface of the reconstructed tympanic membrane

For the patients with pars tensa cholesteatoma and adhesive otitis media, the lesions adhered to the tympanic cavity were stripped off (Fig. 6a, b). Next, cell sheet transplantation around the stapes was performed using the syringe probe device (Fig. 6c), and a tympanic membrane was fashioned using the cartilage/cell sheet hybrid (Fig. 6d, e). Recurrence of tympanic membrane adhesion was prevented by grafting the cell sheet around the stapes and to the rear surface of the newly fashioned tympanic membrane. Figure 6f illustrates the procedure for the cell sheet transplantation in the patients with pars tensa cholesteatoma and adhesive otitis media.

### Patient care and examination

Similar to the procedure after conventional tympanoplasty, the closure of each patient’s wound site was confirmed every day during hospitalization, and the appropriate treatment was administered. Similar to conventional tympanoplasty, hospitalization lasted 6 days and gauze inserted into the ear canal was removed on day 7. Thereafter, the patient came to the hospital once every 2 to 4 weeks as an outpatient and the tympanic membrane was regularly inspected in order to confirm the absence of recurrence. Blood tests (complete blood count and blood chemistry) were performed one month after the transplantation in order to confirm the absence of abnormal changes in test parameters. 1 month, 6 months and 1 year after the surgery, pure-tone audiometry and photography of the tympanic membrane were performed. Evaluation of postoperative hearing improvement was performed using the hearing at 6 months postoperation at the shortest. The evaluation was performed in accordance with the guidelines of the American Academy of Otolaryngology Head and Neck Surgery (AAO-HNS).^37^ The postoperative average hearing level was calculated according to performance using the four-tone average of 0.5, 1, 2, and 3 kHz tests. Postoperative temporal bone CT examinations were conducted after 3, 6, and 12 months.

## Electronic supplementary material

Supplementary Information
