# Supplementary figures and images for: Middle ear mucosal regeneration by tissue-engineered cell sheet transplantation
Source: NPJ Regen Med. 2017 Mar 24;2:6. doi: 10.1038/s41536-017-0010-7 (PMC5665617; doi:10.1038/s41536-017-0010-7)

# Supplementary Figure 1

Case 2

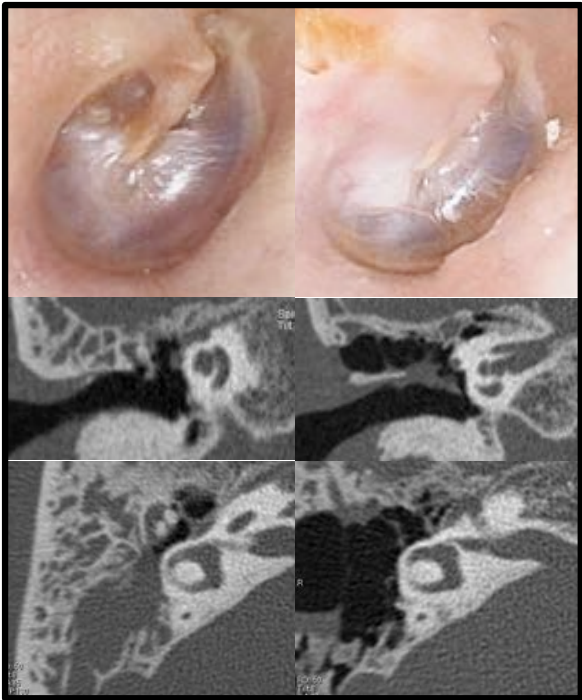

Case 3

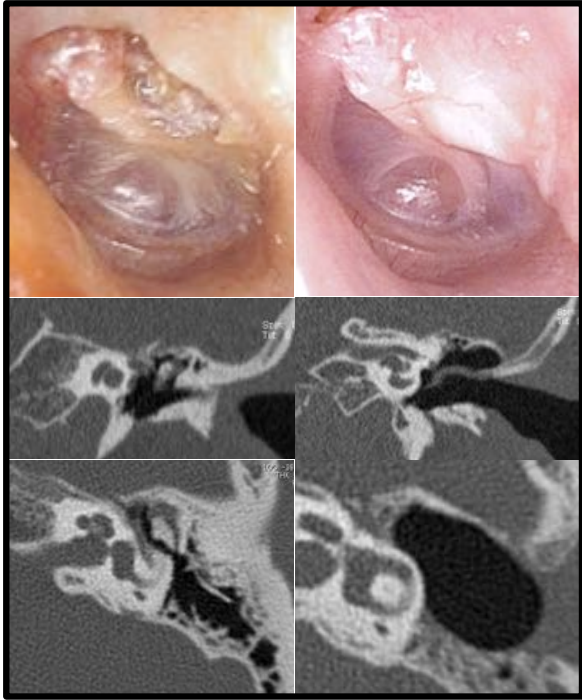

Case 4

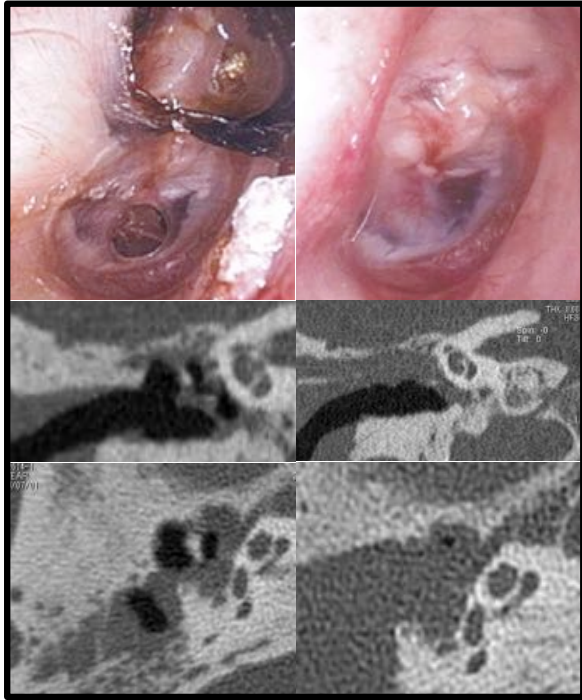

Case 5

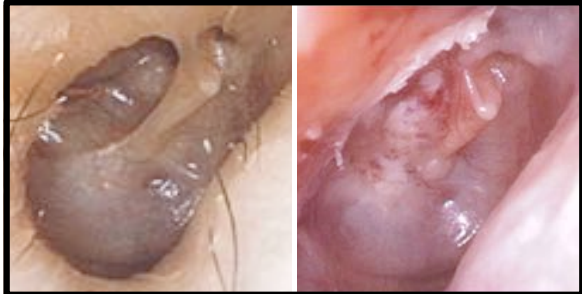

Supplement: Supplementary file 1 — Supplementary Information [file 41536_2017_10_MOESM1_ESM.pdf]
